# Combined approach versus single Henry approach for fixation of die-punch distal radius fractures: a retrospective study

**DOI:** 10.1186/s12893-023-02047-x

**Published:** 2023-06-24

**Authors:** Zisheng Xu, Yuqing Liang, Guobo Geng, Weidong Mu, Peng Xu

**Affiliations:** 1 Shandong Provincial Hospital, Shandong University, Jinan, China; 2 Shandong Provincial Hospital Affiliated to Shandong First Medical University, Jinan, China

**Keywords:** Distal radius fracture, Die-punch fracture, Volar locking plate, Combined volar and dorsal approach

## Abstract

**Background:**

Distal radius fracture (DRF) is one of the most common orthopaedic-related traumas. DRF patients with die-punch fractures have a higher risk of loss of reduction, poorer functional outcomes, and increased risk of complications even after open reduction and internal fixation (ORIF). According to the three-column theory, the lunate fossa is an important part of the intermediate column for load bearing. When the distal radius fracture involves the lunate fossa, adequate anatomical reduction can have an important impact on the prognosis of wrist function. Herein, we used the combined volar and dorsal approach, and the dorsal approach was used to assist in bone grafting or dorsal plate fixation in reducing fractures. We compare the combined approach versus the Henry approach for the fixation of die-punch distal radius fractures.

**Methods:**

We reviewed patients who were admitted for surgery for die-punch fractures from January 2016 to June 2021. The patients were followed-up after surgery to measure and evaluate their Gartland–Werley wrist score, wrist range of motion (ROM), and follow-up imaging data.

**Results:**

There were 21 patients in the volar locking plate (VLP) group and 10 patients in the combined approach group. The majority of fractures in the VLP and combined approach groups were AO B and C fractures, respectively. The cause of injury and AO fracture classification showed significant differences between the two groups, and there was no difference in age or sex between the two groups. There was no significant difference in ROM between the two groups, but the VLP group presented a better Gartland–Werley score and volar tilt angle, and the combined group presented better maintenance in radial height and articular congruity.

**Conclusions:**

Reduction through the combined palmar and dorsal approach supplemented by bone grafting or dorsal plate fixation is an effective method for the treatment of die-punch distal radius fractures, which provides a new option for the treatment of die-punch fractures.

## Introduction

Distal radius fracture (DRF) is one of the most common traumas in orthopaedics. In young people, the aetiology of DRF is typically due to high-violence injuries such as falls from heights; however, low-energy injuries are more common in middle-aged and elderly patients. The traditional treatment method is manual reduction supplemented by plaster or cast. However, intra-articular fractures and extra-articular comminuted fractures often cannot achieve good reduction and effective fixation [1]. Therefore, early open reduction and internal fixation (ORIF) is essential for these cases. In patients with intra-articular comminuted fractures such as AO type C3, even after ORIF treatment, there remains a higher risk of loss of reduction, poor functional outcomes, and increased risk of complications [2, 3], which cannot be ignored by orthopaedic surgeons.

Die-punch fractures are a special category of intra-articular fractures of the distal radius, which were first described by Scheck [4] in follow-up cases wherein a fracture fragment separated from the dorsal medial lunate fossa. Per the present guidelines, clinical practice refers to intra-articular fractures of the distal medial column as die-punch fractures. Rikli et al. [5] proposed the theory of the biomechanical structure of the distal three-column of the radius, highlighting the importance of the lunate fossa as an important part of the middle column for load-bearing. The lunate surface of the distal radius covers 46% of the contact area of the entire wrist joint [6]. The sensor tests in vivo and in vitro also prove the importance of the middle column for the transmission of force loads [7]. Therefore, when the distal radius fracture involves the lunate fossa, adequate anatomical reduction can have an important impact on the prognosis of wrist function.

At present, the main surgical treatment for DRF is open reduction and internal fixation by using a volar locking plate (VLP), which allows early movement, thus effectively avoiding wrist stiffness and reducing pin-tract infection. It can also avoid the wear of the dorsal plate on the extensor tendon. However, in our assessment, although some patients with die-punch fractures on long-term follow-up showed recovery of satisfactory wrist function and return to life and work, the loss of radial height and poor reduction of articular surface bone fragments cannot be ignored when reviewing the imaging results. Therefore, in this study, we used the combined volar and dorsal approach to treat these fractures. The dorsal approach was used to assist in reducing fractures. If necessary, bone grafting and dorsal plate or K wire fixation combined with VLP implantation were used. This study systematically reviewed the efficacy of the conventional volar approach and the combined approach for patients diagnosed with die-punch fractures in Shandong Provincial Hospital, China. The scope of the study includes the following: (1) to measure palmar inclination, ulnar declination, radial height, and step-off of patients’ review imaging data before and after surgery; (2) to use a goniometer to measure wrist range of motion (ROM); (3) to ask patients to cooperate in finishing the wrist function rating scale (Gartland–Werley scoring system); and (4) to evaluate the feasibility of this surgical method via long-term postoperative complications.

## Materials and methods

### Patients and inclusion criteria

This is a retrospective study. We retrospectively collected data from patients diagnosed with DRF between January 1, 2016, and June 1, 2021.

The inclusion criteria were as follows: (1) age ≥ 18 years; (2) die-punch distal radius fracture diagnosed by electronic medical records and clear radiographic evidence; (3) traumatic fracture; and (4) follow-up duration ≥ 1 year. The exclusion criteria were as follows: (1) age < 18 years; (2) pathological fractures or fatigue fractures due to any other nontraumatic factor; (3) patients unwilling to comply with follow-up after surgery; and (4) subacute fractures or fractures with delayed presentation (> 21 days).

### Surgical method

After induction of general brachial plexus anaesthesia, the patient wa placed in the supine position on an operating table equipped with a radiolucent hand table, the affected limb was abducted, and a tourniquet was placed on the upper middle and upper third of the proximal upper arm.

The conventional surgical method includes the volar Henry approach, which entails incising the skin and fascia layer, entering between the brachioradialis muscle and the flexor carpi radialis muscle, separating the radial artery and protecting it, pulling it laterally to expose the pronator quadratus muscle and elevating it to expose the volar surface of the distal radius. The volar capsule or ligamentous insertions were not released. The radial styloid and medial column volar fragments were reduced and secured to the shaft with Kirschner wires. A VA-LCP plate (VA-LCP Two-Column Distal Radius Plate, Depuy Synthes) was placed on the volar side, the gliding hole of the proximal end was fixed with a cortical bone screw, and a Kirschner wire was inserted at the distal end to help maintain the position of the plate. The position of the plate and reduction of the fracture were confirmed by fluoroscopy. If the radial inclination is not good, the plate can be used to help reduction. First, the proximal cortical bone screw was loosened, and distal screws were applied. Then, the proximal cortical bone screw was tightened. At this time, the distal collapsed articular surface can be reduced.

**Fig. 1:**
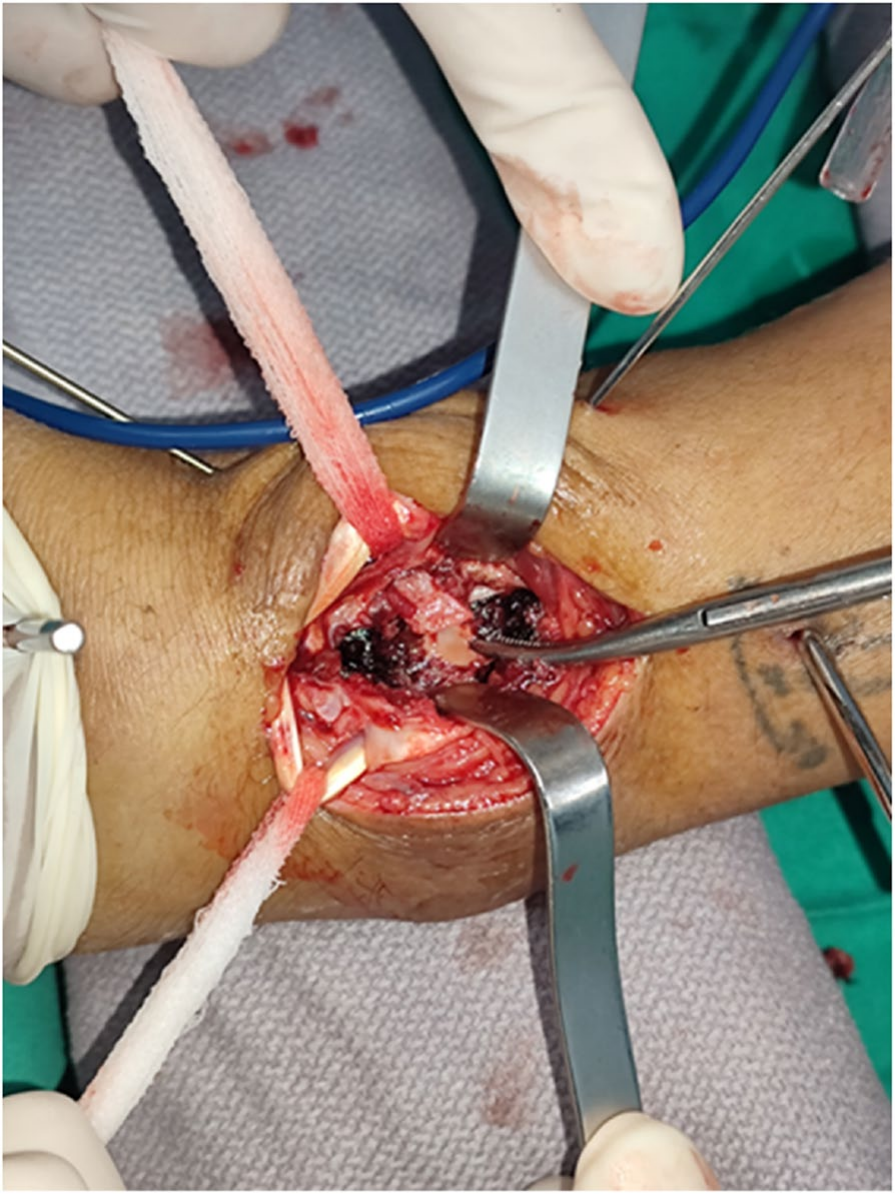
Auxiliary dorsal approach for reduction. After incision of the joint capsule, bone fragments were seen directly

If the articular surface was compressed or the intra-articular bone fragment was still displaced, the distal end of the plate screw was temporarily fixed with 1–2 shorter screws to maintain the length of the radius and the position of volar fragments. Then, a dorsal incision was made along the Lister tubercle. The distal end crosses the radiocarpal joint line and ends at the 1 cm proximal end of the base of the second metacarpal carpal joint, and the proximal end extends 3–4 cm along the radial trunk. The intermediate column was exposed through the bottom of the third extensor space. The extensor retinaculum was severed along the extensor longus tendon and then released and protected. The distal end of the extensor retinaculum was cut in a "V" shape so that the distal end of the extensor longus tendon can maintain its original path and later cover the plate as a soft tissue flap. Peeling along the periosteum revealed the intermediate column, and peeling along the second extensor compartment revealed the dorsal side of the distal radius. A limited incision of the joint capsule was made along the dorsal margin of the radius to observe the surface of the joint, and the compressed or collapsed intra-articular fracture fragment was exposed and elevated (Fig. 1). If necessary, bone defects after reduction can be treated with allogeneic bone. After reconstructing the articular surface, the dorsal fragments were fixed with a dorsal plate or Kirschner wires. Then, we went back to the volar incision, and the longer screws of the distal VLP were applied and finished the final fixation. The surgical incisions were closed layer by layer by suturing. Finally, we used a short-arm plaster splint for immobilization in the first week and then let patients begin gentle active exercises.

**Fig. 2:**
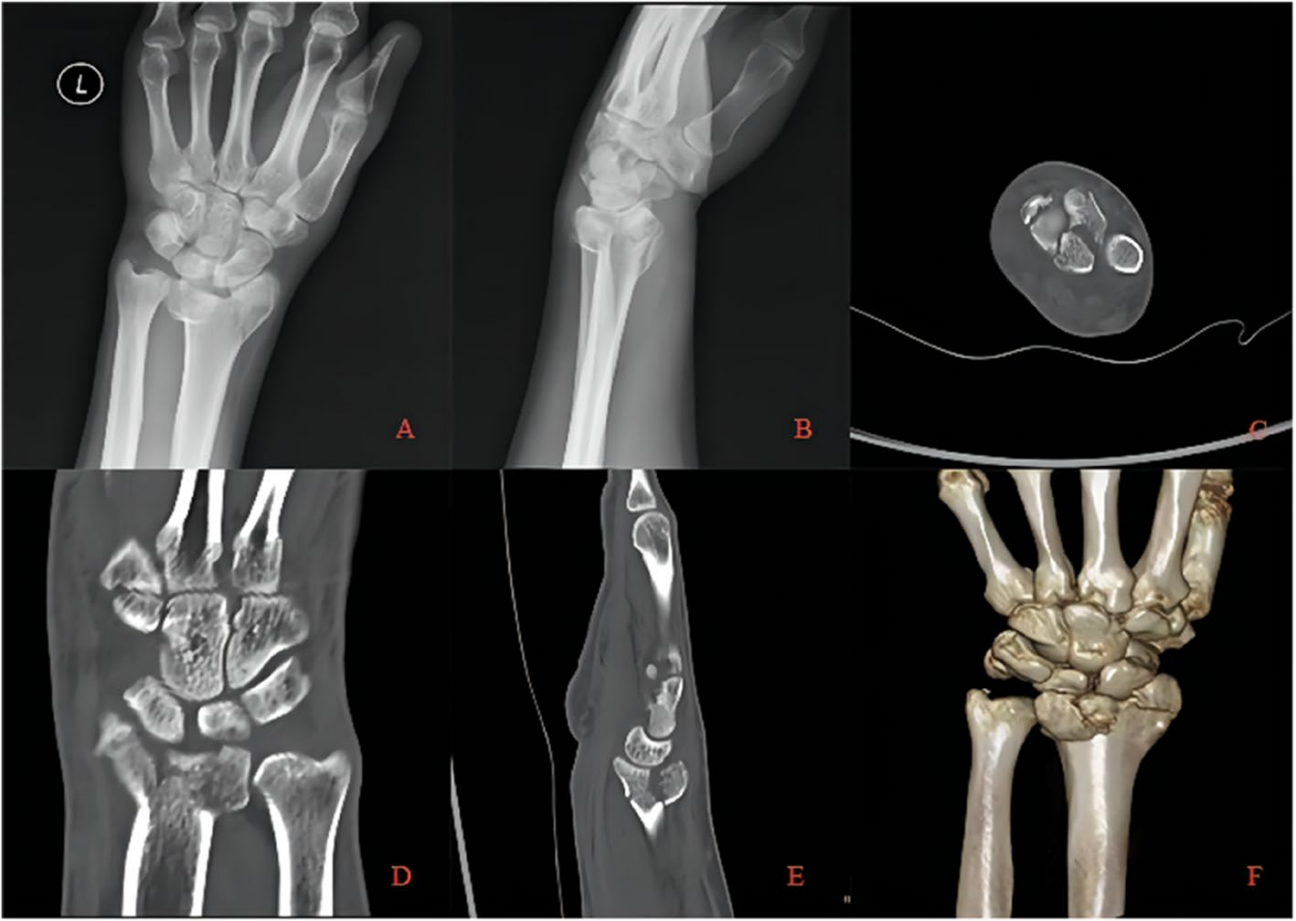
A 38-year-old patient with die-punch DRF. Radiography data showed a die-punch fracture of the left distal radius and a fracture of the articular surface of the intermediate column with obvious displacement. **A** AP radiography; **B** Lateral radiography; **C** Axial view of the articular surface of the radius. The fractures involved the articular surface of the intermediate column and lateral column; **D** Coronal plane, collapse of the lunate articular surface is obvious; **E** Sagittal plane, bone fragment shifted to the dorsal side; **F** Three-dimensional reconstruction of the CT image

**Fig. 3:**
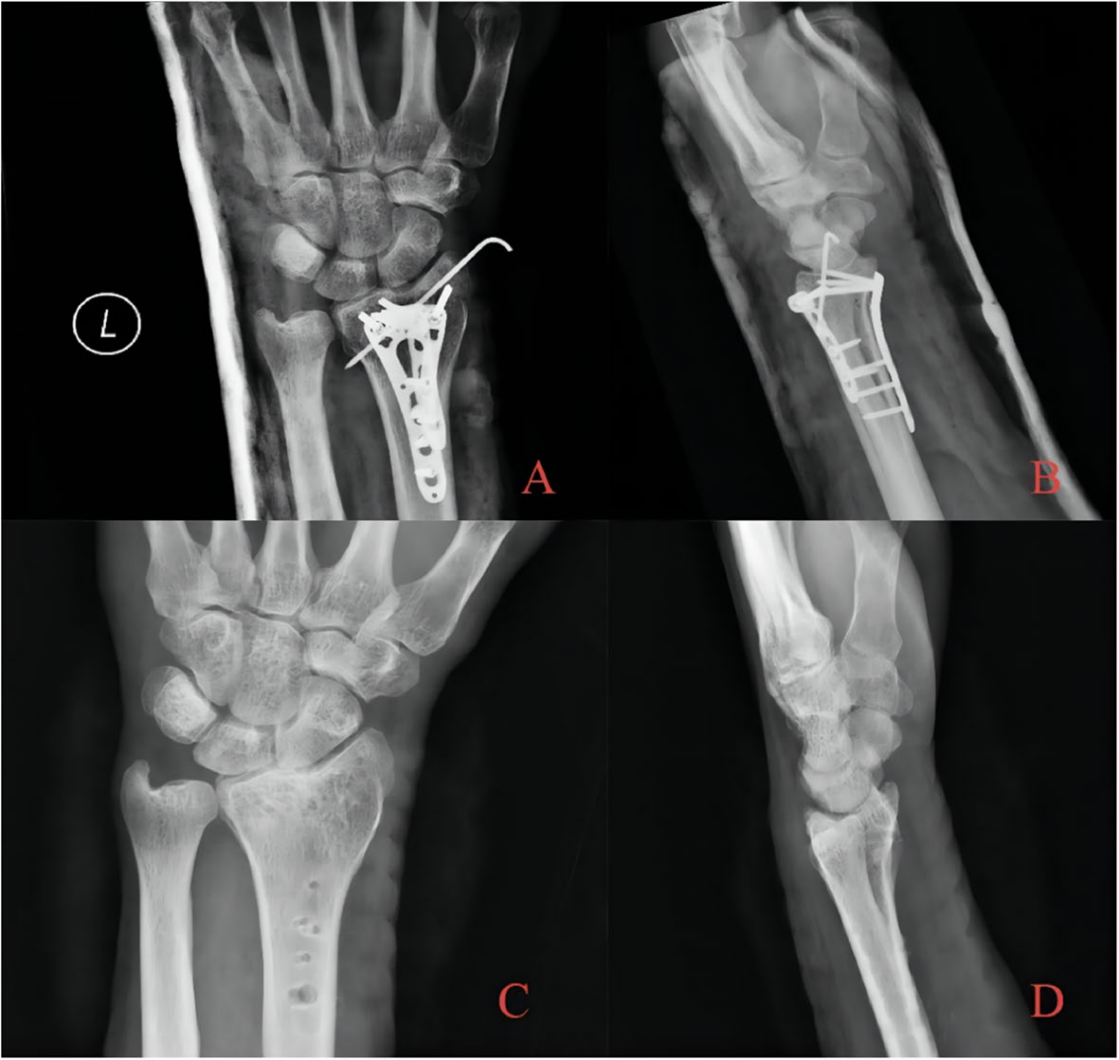
After satisfactory reduction, locking plates were used on both the dorsal and palmar sides. Kirschner wires for auxiliary fixation were retained for 8 weeks and were removed afterwards. The surgical results were good. The long-term follow-up after 30 months showed that the height of the articular surface was well maintained, and there was no obvious collapse of the articular surface. The internal fixator was removed for patient **A** Immediate postoperative AP radiography; **B** Immediate postoperative lateral radiography; **C** AP radiography after removal of internal fixation with follow-up over 30 months. Radial height was well maintained, and the articular surface was congruent in general; hence, no obvious articular step-off developed. **D** Lateral radiography after removal of internal fixation

**Fig. 4:**
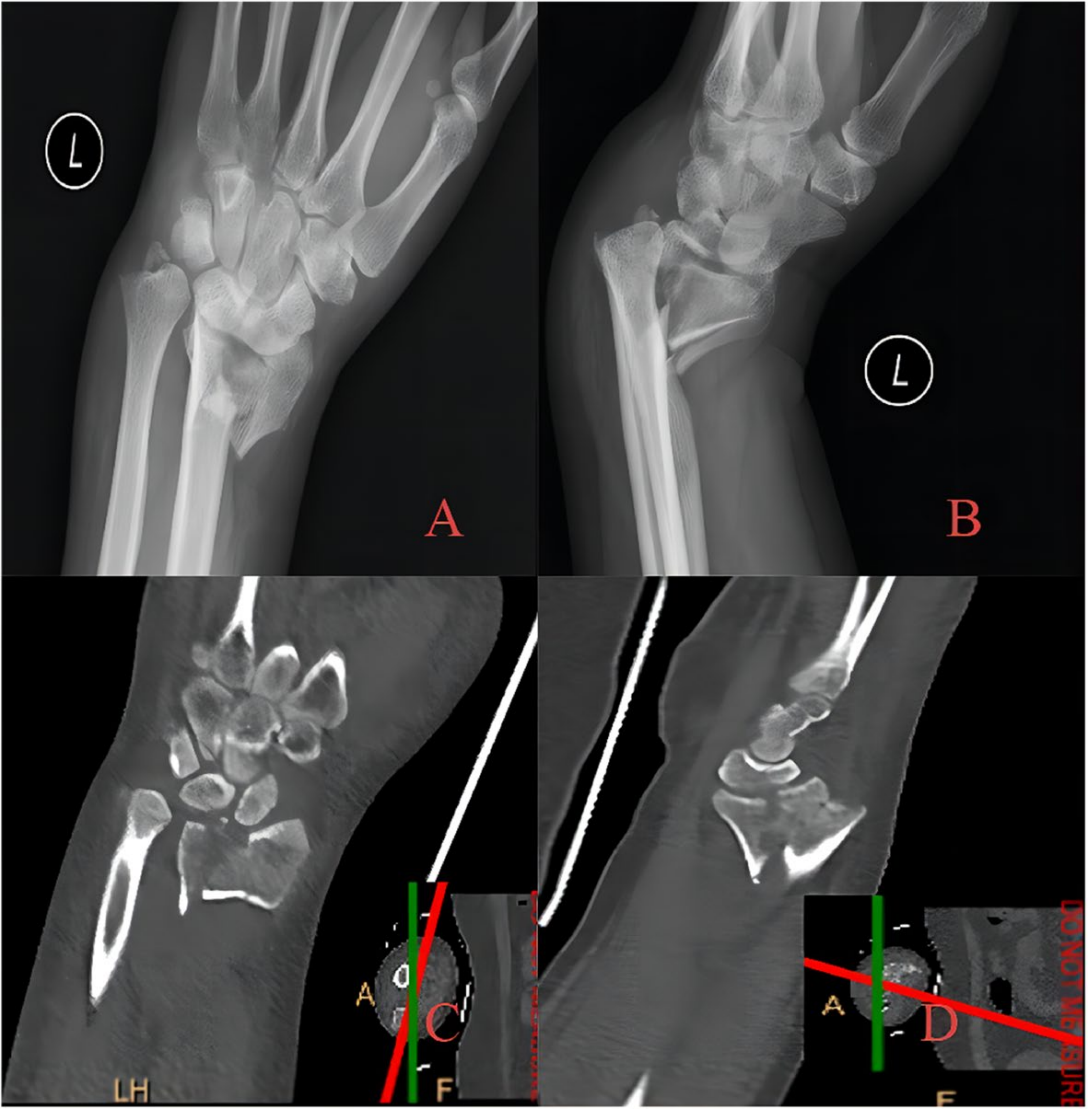
A 27-year-old patient with die-punch DRF. Radiographic examination showed that the lunate surface of the wrist joint was split longitudinally and collapsed downwards, and the fracture fragment was displaced to the palmar side. **A** AP radiography showed that the epiphysis and articular surface were involved; **B** Lateral radiography showed fracture; **C** Axial view of the articular surface of the radius. Fracture involving articular surface of intermediate column and lateral column are noticed; **D** Coronal plane, collapse of the lunate articular surface is obvious; **E** Sagittal plane, bone fragment shifted to dorsal side **F** Three-dimensional reconstruction of CT image

**Fig. 5:**
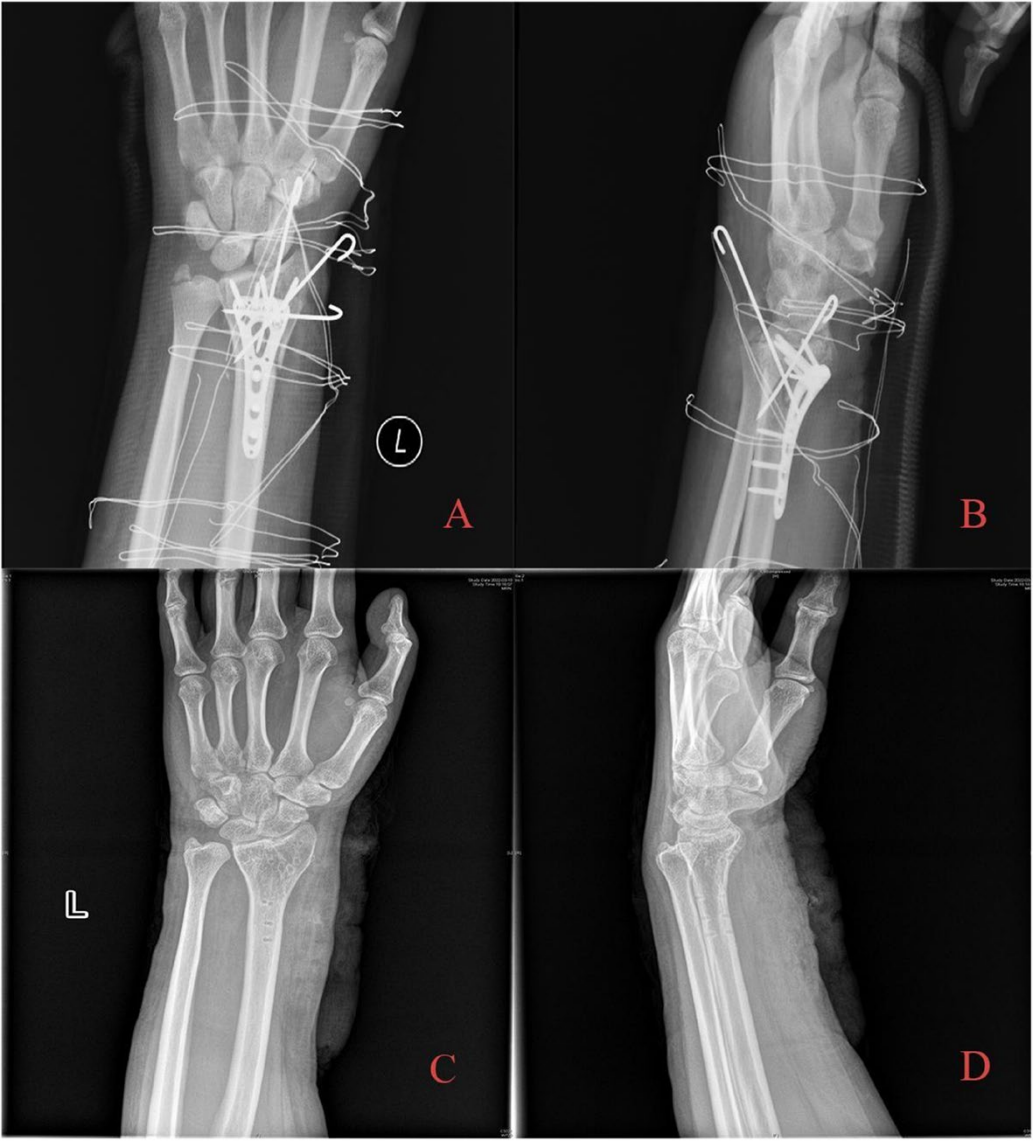
During the operation, it was found that there was a bone defect at the fracture end after indirect reduction. Allogeneic bone grafting was performed, Kirschner wire was used to fix the free bone fragment, and VLP was used to fix the fracture. **A** Immediate postoperative AP radiography; **B** Immediate postoperative lateral radiography; **C** AP radiography after 18 months of follow-up. Radial height was well maintained, and the articular surface was congruent in general; no obvious articular step-off developed. **D** Lateral radiography after removal of internal fixation

Two typical die-punch fracture cases treated with the combined approach are demonstrated in Figs. 2, 3, 4 and 5. Detailed information on patients and imaging is illustrated in the figure legends.

### Radiologic evaluations

Anteroposterior (AP) and lateral position radiographs of the affected wrist joint were used to measure and evaluate the wrist joint during outpatient follow-up. The immediate postoperative radiographs and the 1-year follow-up radiographs of the patients were obtained. Palmar inclination, ulnar declination, radial height difference, and joint regression were measured in radiographs. All complications were reported in the interview, including infection, plate loosening, internal fixation failure, nerve injury, and local pain.

### Clinical assessment

#### Gartland–Werley score

The Gartland–Werley score combines items such as residual deformity, competent sensation, objective assessment, and complications. Lower scores represent better functionality, and higher scores represent worse functionality.

#### Wrist range of motion (ROM)

Using a protractor, the palmar inclination, dorsal extension, ulnar deviation, radial deviation, pronation, and supination of the wrist joint were measured and compared between the groups.

### Statistical analysis

Patient characteristics, including age, palmar inclination, ulnar declination, wrist ROM, and radial height difference, were continuous variables and presented as the mean and standard deviation (SD). Based on the normal distribution of the included variables, the two study groups were compared using the *t* test or the Mann‒Whitney U test. Categorical variables such as sex, injury mechanism, and fracture type were expressed as frequencies and percentages. Pearson’s chi-square test or Fisher’s exact test was used to analyse data for categorical variables. The significance threshold was set at *P* = 0.05. Statistical analysis of each data point was performed using SPSS 26.0 software (IBM Corporation, Armonk, NY, USA).

## Results

### Demographic details of patients in the study

From June 2016 to June 2021, based on the abovementioned inclusion and exclusion criteria, 31 patients with complete data agreed to undergo postoperative follow-up and were included in this study for analysis. All patients were followed-up for more than 1 year. Among them, 21 patients were treated with VLP through a single Henry approach, and 10 patients were treated with a combined approach for die-punch fractures. There were 11 women and 10 men in the VLP group (mean age: 42.71 ± 15.50 years). In the combined approach group, there were 4 women and 6 men (mean age: 46.20 ± 13.40 years). In the VLP group, falls and traffic accidents were the main causes of injury, while in the combined approach group, falls from heights and traffic accidents were the main causes of die-punch fractures. The majority of fractures in the VLP group were AO type B and C1 or 2 fractures, whereas more patients in the combined approach group had AO type C3 fractures. The cause of injury and AO fracture classification showed significant differences between the two groups, and there was no difference in age or sex between the two groups. The detailed data of the patients are shown in Table 1.

**Table 1:** Demographic details of patients in the VLP group and combined approach group

	VLP group	Combined approach group	*P* value
Age	42.71 ± 15.50	46.20 ± 13.40	0.547
Male (percentage)	10(47.6%)	6(60%)	0.795
Mechanism			0.028
Fall from height	2	5	
Fall from standing	13	2	
Traffic accident	6	3	
AO classification			0.021
B	16	3	
C	5	7	

### Functional scores and wrist ROM between the VLP group and combined approach group

There was no significant difference between wrist pronation, supination, palm tilt, dorsal extension, ulnar deviation, and radial deviation in the two treatment groups. However, the Gartland–Werley wrist joint score was significantly better in the conventional VLP group than in the combined approach group (*P* < 0.001). The detailed data are shown in Table 2.

**Table 2:** Functional results between VLP group and combined approach group

Parameters	VLP group	Combined approach group	*P* value
Pronation (deg)	81.31 ± 5.30	71.78 ± 13.37	0.054
Supination (deg)	81.98 ± 3.91	81.88 ± 5.81	0.957
Extension (deg)	78.36 ± 5.76	74.79 ± 7.20	0.148
Flexion (deg)	68.44 ± 15.74	63.40 ± 16.97	0.423
Ulnar deviation (deg)	34.53 ± 6.99	29.77 ± 5.62	0.07
Radial deviation (deg)	9.37 ± 2.88	11.47 ± 5.86	0.306
Gartland–Werley score (points)	1.86 ± 1.88	4.8 ± 1.62	< 0.001

### Radiographic parameters between the VLP group and combined approach group

There were significant differences in imaging outcome measures between the two groups. The recovery of palm inclination in the combined approach group was worse (*P* = 0.004). However, in the maintenance of radial height, the combined dorsal palmar approach showed better results (*P* < 0.001).

The step-off of the articular surface is currently clinically recognized as a parameter that has a greater relationship with the recovery of wrist joint function. Unexpectedly, the combined palmar and dorsal approach group had a smaller step-off value in cases of worse wrist function scores, and the overall wrist joint maintained better consistency. The detailed data are shown in Table 3.

**Table 3:** Differences in radiographic parameters between the VLP group and combined approach group

Parameters	VLP group	Combined approach group	*P* value
Volar tilt (deg)	11.50 ± 3.46	7.30 ± 3.54	0.004
Radial inclination (deg)	21.38 ± 5.13	19.20 ± 4.80	0.269
Radial height difference value(mm)	0.164 ± 0.07	0.053 ± 0.02	< 0.001
Articular step-off (mm)	2.59 ± 0.57	1.70 ± 0.31	< 0.001

## Discussion

Die-punch DRF fractures are intra-articular fractures, and most patients do not receive satisfactory intraoperative indirect reduction. In the long term, this joint incongruity may further lead to chronic complications such as traumatic arthritis and active wrist pain and reduce the quality of life of patients.

Most distal radius fractures, even AO type C3, can be treated by a single Henry approach, but for special circumstances such as surface collapse, effective reduction cannot be obtained by traction and clamps because it is difficult to expose the articular surface of the distal radius on the volar incision. Auxiliary dorsal incision can effectively solve this problem. Through the dorsal incision, the surgeon can cut the joint capsule to look at the articular surface easily and reduce the collapsed or compressed articular surface directly, as well as effective dorsal fixation and bone grafting. All these measures can help prevent postoperative loss of reduction and fixation failure.

Some scholars have noted that compared with the dorsal plate, the volar plate may have a higher incidence of fracture collapse, especially when patients suffer from dorsally displaced fractures [8]. Even if the joint is successfully reduced in ORIF, the loss of reduction in the long term is common in patients with the die-punch classification. Loss of reduction has been reported in 6.7–9.8% of patients treated with VLPs in DRF [9]. However, in a comparative study of DRFs by Zhang et al. [10] Regarding involvement of the lunate fossa, 19% of patients with lunate fossa involvement had joint regression, compared with only 4.2% of patients in the control group. In another study published by the author’s team, the articular surface step-off rate of VLP patients also reached 12% [11]. Earp used a single volar plate for the treatment of AO23-C3 distal radius fractures and found that 62.5% (5/8) of cases of postoperative significant loss of reduction occurred in fractures involving the lunate facet joint [9]. The high rate of loss of reduction in die-punch fractures and the proportion of articular surface step formation in the overall DRF may be related to the functional structure of the high axial load at the distal lunate fossa of the radius. In recent years, articular surface regression has been proven to be an important risk factor for long-term traumatic arthritis of various types of fractures [12, 13]. In recent studies, scholars have taken the articular surface step-off by > 2 mm as a risk factor for articular surface instability and even long-term traumatic arthritis [11, 14, 15].

For VLP, its short-term functional rehabilitation [15] and low rate of stiff joints and worn tendons are important advantages compared with the dorsal plate, external fixation, and other fixation methods. However, as cited above, fixation of DRFs involving the lunate articular surface with only the VLP approach tends to have a high rate of long-term loss of wrist reduction. The extended flexor carpi radialis (FCR) approach is also one of the alternate surgical approaches for die-punch fracture [16]. Compared with the traditional Henry approach, this surgical method can better reveal the fragments and facilitate reduction for surgeons. However, it severely peels off the periosteum of the distal radius and its surrounding soft tissue. The exposure of the articular surface may not be satisfactory compared to the dorsal approach, which has been proven earlier [17].

In one study of AO23-A2 and AO23-A3 DRFs, patients with radial loss had worse Mayo wrist scores [18]. Therefore, maintenance of articular surface consistency is even more important in patients with Type B and C fractures. The use of VLPs alone may not be sufficient in the die-punch classification of collapsed fractures involving the lunate surface. According to the literature reports, in some patients with AO type C fractures, the fragments tend to collapse to the opposite side if the plate is placed on one side [19]. This may also partly explain the high rate of loss of reduction during the volar approach in most of the literature, including those cited above. This is understandable given the current maturity of VLP technology and the significant disadvantage of dorsal plates. However, considering how to maintain the height of the articular surface, we believe that it may be meaningful to explore the advantage of Henry’s approach combined with the dorsal incision in clinical treatment.

After reduction of the bone fragment through the combined dorsal approach during the operation, we implanted or allogeneic bone for the possible residual bone defect. In patients who are still unstable after volar plate placement, we may consider retaining the K-wire to stabilize the bone fragment or reinserting the dorsal plate. In the long-term follow-up imaging data, the overall preservation of radial height and articular surface consistency in this group of patients was ideal, even compared to patients with significantly less damage, which was unexpected.

Although many studies have analysed and summarized the high reduction loss rate of die-punch fractures, relevant clinical studies on the treatment of the reduction loss rate are still lacking. In our study, we found that a dorsal approach with assisted fixation of bone grafts can achieve good alignment. We noted that the main complications were local numbness of the wrist (2/31) and wrist discomfort because of bad weather, such as rainy days (3/31); no infection, median nerve injury, or tendon rupture were found, although extensor complications are major impediments to performing dorsal plates. However, the Gartland–Werley wrist joint function score showed that the final follow-up results of patients in the traditional volar approach group were better, and the recovery of radial inclination in the traditional group was also better in the imaging data. We believe that these statistically significant differences are mainly because of more frequent use of the combined volar and dorsal approach in AO type C fractures (*P* = 0.021), whereas type C DRFs tend to result from more severe injuries; to some extent, this affects the prognosis of patients. This is likely how the combined approach group can achieve better radial height and joint consistency results that are more clinically meaningful.

In previous studies, some scholars reported that even displaced DRFs can be fixed with a single volar approach to ensure an excellent prognosis [20]. However, the postoperative evaluation index did not mention the radial height or articular surface step-off follow-up. Uncertainty of long-term articular surface congruity may be a predisposing factor for later traumatic arthritis in patients. Moreover, researchers have also discussed the possibility of combined volar and dorsal approaches in the treatment of DRFs in recent years. Jung et al. suggested that after fixation of VLPs in unstable DRFs, an additional dorsal approach could be used distally to remove intra-articular or migrated dorsal fragments and restore joint consistency [21]. Medlock [22] also applied the dorsal palmar plate in the treatment of C-type DRFs and achieved good results while avoiding complications such as tendon wear and rupture caused by the dorsal plate approach.

This study has some limitations: the patient follow-up endpoint was set at 1 year, and there were no longer-term follow-up results to comment on additional complications. Since there was no interobserver reliability in our fracture classification process, there might be a potential bias. Owing to differences in the frequency of early and mid-term follow-up and loss to early-time follow-up, their results were not included in the common evaluation. In addition, it is an unavoidable fact that a complicated fracture classification tends to lead us to take a different surgical approach, which may give rise to a bias in the choice of treatment approach. Complications of tendon irritation with the dorsal approach are always nonnegligible in long-term clinical outcomes as well.

## Conclusion

Combined reduction using the combined palmar and dorsal approach, supplemented by bone grafting or dorsal plate fixation, is an effective method for the treatment of die-punch DRFs, which has the potential to contribute to guidelines for future treatment decisions for this fracture type.

## Data Availability

The datasets of the current study are available from the corresponding author upon reasonable request.

## Abbreviations

DRF: Distal radius fracture
ORIF: Open reduction and internal fixation
VLP: Volar locking plate
ROM: Range of motion
AP: The anteroposterior
SD: Standard deviation
